# Performance evaluation of methods for detecting enteric viruses in meat products

**DOI:** 10.1007/s42770-026-02058-9

**Published:** 2026-08-18

**Authors:** Lucas Zanchetta, Paula Rogovski, Henrique Borges da Silva Grisard, Beatriz Pereira Savi, Catielen Paula Pavi, Sabrina Carvalho Suominsky, Giulia Von Tönnemann Pilati, Gislaine Fongaro

**Affiliations:** Laboratory of Applied Virology, Department of Microbiology, Immunology and Parasitology, Federal University of Santa Catarina, Florianópolis, 88040- 900 Brazil

**Keywords:** Foodborne viruses, Hepatitis E virus, RT-qPCR, Food safety surveillance

## Abstract

Meat products represent important vehicles for foodborne viral transmission, yet standardized detection methodologies for these complex matrices remain limited. This study systematically compared three viral recovery methods across nine meat products representing chicken, pork, and processed deli meat matrices: polyethylene glycol 6000 (PEG) precipitation, glycine buffer-chloroform (GBC) phase separation, and β-mercaptoethanol (B-M) chaotropic lysis. Using Murine Norovirus 1 (MNV-1) and Murine Hepatitis Virus 3 (MHV-3) as process controls, recovery efficiency was assessed by RT-qPCR. The B-M method demonstrated the most consistent performance, successfully detecting MNV-1 across all tested matrices and significantly outperforming PEG and GBC for MNV-1 in chicken and pork. Applying the B-M method to 25 commercial retail products revealed the genetic material of Hepatitis E virus (HEV) in four samples and Porcine Circovirus type 2 (PCV-2) in one sample, exclusively within pork products. These findings provide critical comparative performance data for method selection in viral surveillance of meat and document the occurrence of HEV and PCV-2 genetic material in commercial pork products from Santa Catarina, Brazil, highlighting a significant food and agricultural biosafety concern in this major meat-producing and exporting state.

## Introduction

 Foodborne illnesses represent a substantial global public health burden, arising from diverse etiological agents including bacteria, viruses, parasites, and chemical or physical contaminants [1]. Among these, enteric viruses have emerged as leading causes of foodborne outbreaks, with significant implications for human health and economic costs [2, 3]. The epidemiological importance of foodborne viruses stems from their unique biological characteristics: low infectious doses (often fewer than 100 viral particles), high environmental stability, and efficient person-to-person transmission [1, 2, 4]. Non-enveloped enteric viruses, in particular, exhibit resistance to environmental stressors, including temperature fluctuations, pH extremes, and common sanitation procedures, enabling viral persistence in contaminated foods for up to four weeks post-contamination [5, 6].

Meat products represent an important food safety concern of increasing relevance due to their global production scale, complex distribution chains, and documented role as vehicles for viral transmission [7, 8]. Viral contamination of meat can occur through multiple pathways, including cross-contamination from infected food handlers, inadequate hygiene during processing or storage, environmental contamination during slaughter, and direct zoonotic transmission through infected animal tissues [7]. Human Norovirus (NoV), the predominant cause of non-bacterial gastroenteritis worldwide, contaminates meat primarily through infected handlers or environmental sources [2, 7]. Hepatitis A virus (HAV) follows similar anthroponotic transmission routes, while Rotavirus A (RVA) represents an emerging foodborne pathogen in meat products [2]. In contrast, Hepatitis E virus (HEV) poses a significant zoonotic threat, with genotypes 3 and 4 directly transmitted through consumption of contaminated pork, wild boar, and deer products [8]. Beyond human pathogens, animal-specific viruses such as Rotavirus C (RVC), Porcine Adenovirus (PAdV), and Porcine Circovirus (PCV) serve as valuable indicators of fecal contamination and process control markers in meat processing environments [5, 7].

Despite the recognized importance of viral surveillance in foods, standardized detection methodologies remain limited in scope. ISO 15216-1:2017 [9], the international reference standard for viral quantification, specifies procedures for NoV and HAV detection in bivalve mollusks, leafy vegetables, berries, and water using Reverse Transcription Quantitative Polymerase Chain Reaction (RT-qPCR) but notably excludes meat products [9]. This exclusion reflects the unique analytical challenges posed by meat matrices, which contain high concentrations of proteins and lipids that interfere with both viral recovery and nucleic acid amplification [4, 5, 10]. Proteins, particularly heme-containing compounds, can inhibit or denature polymerase enzymes, while lipids interfere with extraction efficiency and may co-precipitate with viral particles during concentration steps [10]. If not effectively removed, these inhibitors lead to underestimation of viral loads or false-negative results [10]. The characteristically low viral load further increases these technical challenges in contaminated meat products, which are often near detection limits yet still sufficient to cause infection, thus necessitating highly sensitive and efficient recovery methods [7, 10].

The absence of validated, standardized protocols for viral recovery from complex food matrices underscores the critical need for systematic evaluation of extraction methodologies. Process control viruses, which are surrogate organisms used to assess recovery efficiency, extraction performance, and detection sensitivity, are essential tools in such evaluations. In this context, the present study systematically compared three viral recovery methods across nine distinct meat products representing diverse compositional profiles (chicken, pork, and processed deli meats). Using Murine Norovirus 1 (MNV-1) and Murine Hepatitis Virus 3 (MHV-3) as process controls, the present study assessed method performance across matrices to identify the most consistent approach for meat applications. The validated method was subsequently applied to screen 25 commercial meat products from Santa Catarina, Brazil (a major meat-producing region) for the presence of NoV, HAV, HEV, RVA, RVC, PAdV, and PCV-2, providing insights into viral contamination prevalence in commercially distributed products.

## Materials and methods

To ensure a thorough assessment of viral recovery across various food compositions, three methodologies were evaluated on nine distinct meat products. These products included three different cuts each of chicken (breast, wings, thigh) and pork (rib chop, belly, ground pork), as well as three types of deli meat (Italian salami, colonial salami, coppa). MNV-1 and MHV-3 were used as controls to determine the recovery efficiency of each method. The spiked concentration of these controls was quantified in genomic copies (GC): 3.0 × 10^11^ GC (3.0 × 10^13^ GC/mL) for MNV-1 and 1.2 × 10^7^ GC (1.2 × 10^9^ GC/mL) for MHV-3. MNV-1 serves as a highly suitable, universally accepted surrogate for human norovirus and other environmentally stable non-enveloped enteric viruses [9]. In contrast, MHV-3, an enveloped murine coronavirus, was included to validate the protocols for the recovery of enveloped viruses of pandemic concern. While enveloped viruses are generally less resilient in food matrices compared to non-enveloped enteric pathogens, the COVID-19 pandemic highlighted a critical necessity for validated methodologies to detect coronaviruses (such as SARS-CoV-2) on food surfaces, packaging, and meat products [11]. Following this evaluation, the most effective methodology was then utilized in a subsequent survey to screen 25 commercial meat products for NoV, HAV, HEV, RVA, RVC, PAdV, and PCV-2.

Viral stocks were propagated in permissive cell lines [12]: MNV-1 in RAW-264.7 cells (murine macrophages, University of Barcelona) and MHV-3 in L929 cells (murine fibroblasts, University of Campinas). Both cell lines were maintained at 37 °C in 5% CO₂. The confluent cell monolayers were inoculated with respective viruses, incubated for 1 h for viral adsorption, and supplemented with fresh maintenance medium until a complete cytopathic effect was observed. Viral particles were released via three freeze-thaw cycles, and lysates were clarified by centrifugation (3,000 ×g, 5 min, 4 °C). Supernatants were stored at -80 °C. Viral concentrations were determined by RT-qPCR using standard curve, and the final stocks contained 3.0 × 10¹³ GC/mL (MNV-1) and 1.2 × 10⁹ GC/mL (MHV-3).

The sausages were of artisanal origin, subjected to State (S.I.E.) or Municipal (S.I.M.) sanitary inspection, and stored at room temperature (~ 25 °C) at the commercial retail level. The raw meat cuts were subjected to State inspection and were maintained under commercial refrigeration (4 °C). All products were purchased between May and June 2024 from local supermarkets and stores in the state of Santa Catarina, Brazil, a region selected for its economic significance in meat export. The refrigerated cuts were purchased as larger, intact pieces of meat. To ensure representativeness, the entire sample was first fragmented into smaller pieces using a sterile scalpel. Subsequently, the samples were processed using the coning and quartering technique [13], which consists of thoroughly homogenizing the fragments and dividing them into quartiles to obtain a representative subsample. The samples were then aliquoted to the specific volumes required for each method and stored at 4 °C for no longer than 24 h until processing.

### Viral recovery methods

#### Polyethylene glycol 6000 (PEG) based method

The Polyethylene glycol 6000 (PEG) method was developed to optimize viral recovery in meat matrices by combining the clarification and elution steps from published methodologies [14, 15] with the concentration step using PEG 6000 adapted from Lewis and Metcalf [16]. A novel initial step, the addition of PBS, was incorporated to improve sample dissolution and facilitate subsequent pH adjustment, which is often difficult in dense matrices. Centrifugation speeds were adjusted to optimize pelleting from these different matrix compositions. Furthermore, a Stomacher-type blender was used for sample processing.

Briefly, 25 g of the sample was combined in a sterile bag with 20 mL of PBS and 10 µL of each MNV-1 and MHV-3 process control. The mixture was then homogenized for 120 s in a Stomacher-type blender. For clarification and elution, the homogenate was transferred to a tube, and the pH was adjusted to 4.5 [14, 15]. Then, samples were homogenized on a rocking platform for 30 min at room temperature and then centrifuged for 15 min at 7,000 ×g at 4 °C for clarification. After discarding the supernatant, the pellet was resuspended in glycine buffer (0.25 M, pH 9.5) and agitated again for 30 min on a rocking platform for elution, then centrifuged at 7,000 ×g for 30 min at 4 °C. Next, PEG 6000 was added to the supernatant to a final concentration of 10%, and the mixture was homogenized on a rocking platform for 2 h at 4 °C [16]. The sample was then centrifuged at 15,000 ×g for 90 min at 4 °C. After centrifugation, the supernatant was discarded, and the pellet was resuspended in 5 mL of PBS. This final solution was collected and stored at -80 °C until nucleic acid extraction.

#### Glycine buffer-chloroform (GBC) based method

The Glycine Buffer-Chloroform (GBC) method was adapted from the work of Moor and collaborators (2018) [17]. The primary modification was the substitution of TRIzol with glycine buffer (0.25 M, pH 9.5). This change was implemented to simplify the workflow, testing if a safer, more common buffer (already in use for the PEG protocol) could be a viable substitute. First, in a sterile bag, 7 g of sample, 7 mL of glycine buffer (0.25 M, pH 9.5), 5 mL of PBS, and 10 µL of each MNV-1 and MHV-3 process control were added. This mixture was then homogenized for 120 s using a Stomacher-type blender. The homogenate was transferred to a new tube, mixed with 1.4 mL of chloroform, and incubated for 10 min at room temperature. Then, the sample was centrifuged again at 10,000 ×g for 15 min at 4 °C. The final supernatant was collected and stored at -80 °C until nucleic acid extraction.

#### β-mercaptoethanol (B-M) based method

The B-M method was adapted from Degenhardt [18]. To improve sample representativeness and homogeneity compared to the original protocol, a pre-homogenization step using a larger sample volume (5 g) was introduced before processing the standard 0.25 g aliquot. Briefly, 5 g of the sample was combined in a sterile bag with 4 mL of RLT lysis buffer (a highly denaturing, guanidine thiocyanate-based buffer) with β-mercaptoethanol (100:1 v/v). The mixture was then homogenized for 120 s in a Stomacher-type blender. Then, the Degenhardt method was employed; 0.25 g of the homogenate was transferred to a 1.5 mL microtube, to which 1 mL of RLT buffer containing β-mercaptoethanol (100:1 v/v) was added along with 10 µL of each MNV-1 and MHV-3 process control. The sample was incubated for 5 min at room temperature and subsequently centrifuged at 10,000 ×g for 20 min at 4 °C. The final supernatant was collected and stored at -80 °C until nucleic acid extraction.

### Genetic material extraction and qPCR/RT-qPCR

Genetic material was extracted from the stored supernatant of each methodology using the RNAdvance Viral XP kit (Beckman Coulter), according to the manufacturer’s specifications. The extracted nucleic acids were diluted tenfold and analyzed in duplicate on a StepOnePlus Real-Time PCR system (Applied Biosystems). For the survey of the 25 commercial meat products, MNV-1 was spiked into all samples prior to processing to serve as a process control, ensuring the success of the extraction protocol across the diverse commercial matrices. RT-qPCR reactions for RNA viruses were conducted using the QuantiNova probe RT-PCR Kit (Qiagen), while qPCR reactions for DNA viruses utilized the TaqMan Universal PCR MasterMix Kit (Applied Biosystems). Standard curves were generated using plasmids with known concentrations that contained cloned sequences of the target viruses. Primers and probes for each virus were selected from previously described methodologies as follows: MNV-1 [19], MHV-3 [20], NoV [21], HAV [22], HEV [23], RVA [24], RVC [24], PAdV [25], and PCV-2 [26]. The standard curves presented a coefficient of determination (R²) ≥ 0.99, an amplification efficiency of > 90% across all independent runs, and a Limit of Quantification (LOQ) established at 10 GC/µL. For the meat products survey, positivity criteria were established based on standard curve analysis and non-template control (NTC) results. A qualitative detection threshold was set at Cq ≤ 40. A sample was considered positive only if concordant amplification below this threshold was observed across both technical replicates, and all NTCs remained negative. This technical replicate concordance requirement, combined with the inherent target specificity of the TaqMan chemistry, served to minimize random single-well artifacts and primer-dimer formation. However, because downstream amplicon confirmation (e.g., sequencing) was not performed, detections at high Cq values are reported cautiously.

### Data analysis

**Fig. 1:**
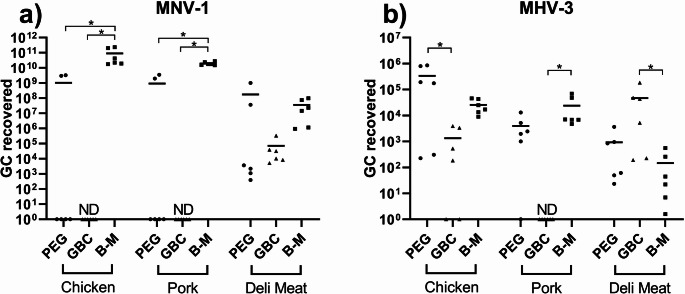
Viral recovery comparison of meat processing methods within each matrix type. Panels show recovery of (**a**) Murine Norovirus (MNV-1) and (**b**) Murine Hepatitis Virus (MHV-3) in absolute genome copies (GC) from chicken, pork, and deli meat matrices. Within each matrix, PEG, GBC, and B-M methods were compared. Each dot represents an individual replicate (*n* = 2 per product, 6 total per matrix). Horizontal bars indicate mean recovery. Brackets with asterisks indicate statistically significant pairwise differences (Dunn’s post hoc test: * *p* < 0.05). ND, Not Detected. Points at 10^0^ represent non-detected samples

Method performance was evaluated using two primary metrics: the recovery rate (mean ± SD) and the absolute GC recovered. For all analyses, undetectable Cqs were treated as zero GC. First, a recovery rate was calculated for each product by dividing the GC quantified by the initial spiked GC quantity. These rates are presented as a mean ± SD for each individual product (*n* = 2 biological replicates) (Table 1) and for the aggregated food matrix (*n* = 6, Table 2). Second, the absolute number of GC recovered was used for all statistical comparisons and graphical visualization. These absolute GC counts were aggregated by food matrix, yielding a total of *n* = 6 independent biological replicates per matrix group (Fig. 1); for visual representation, the replicates that presented zero GC were assigned a nominal baseline value of 10^0^ GC to allow for representation on the logarithmic scale. Statistical analysis was conducted on these aggregated data (*n* = 6). Data normality was first assessed using the Shapiro-Wilk test. As the assumption of normality was not met (*p* ≤ 0.05), differences between the three methodologies within each matrix were analyzed using the Kruskal-Wallis test followed by Dunn’s post hoc test for multiple comparisons. A *p*-value ≤ 0.05 was considered statistically significant. All analyses were conducted in GraphPad Prism 8.0.2 software.

**Table 1:** Mean percent recovery (± standard deviation) of Murine Norovirus (MNV-1) and Murine Hepatitis Virus (MHV-3) from nine different meat products using the PEG, GBC, and B-M methods. Each product was analyzed in duplicate (*n* = 2). ND indicates Not Detected, and ND* indicates recovery < 0.001%

Product	Method	MNV-1 Mean (SD)	MHV-3 Mean (SD)
Chicken wing	PEG	ND	1.525% (± 0.092%)
	GBC	ND	ND*
	B-M	71.745% (± 7.418%)	0.175% (± 0.049%)
Chicken breast	PEG	0.540% (± 0.764%)	ND*
	GBC	ND	ND*
	B-M	6.500% (± 1.075%)	0.370% (± 0.014%)
Chicken thigh	PEG	0.490% (± 0.693%)	6.830% (± 0.339%)
	GBC	ND	0.031% (± 0.004%)
	B-M	10.115% (± 5.424%)	0.090% (± 0.028%)
Rib chop	PEG	0.580% (± 0.820%)	0.005% (± 0.007%)
	GBC	ND	ND
	B-M	7.085% (± 2.864%)	0.060% (± 0.000%)
Pork belly	PEG	ND	0.020% (± 0.000%)
	GBC	ND	ND*
	B-M	6.830% (± 1.485%)	0.490% (± 0.127%)
Ground pork	PEG	0.320% (± 0.453%)	0.075% (± 0.049%)
	GBC	ND	ND*
	B-M	5.310% (± 0.636%)	0.050% (± 0.014%)
Italian salami	PEG	ND*	0.005% (± 0.006%)
	GBC	ND*	0.203% (± 0.189%)
	B-M	0.018% (± 0.021%)	0.001% (± 0.001%)
Colonial salami	PEG	ND*	0.020% (± 0.012%)
	GBC	ND*	0.970% (± 0.694%)
	B-M	0.013% (± 0.010%)	ND*
Coppa	PEG	0.173% (± 0.195%)	ND*
	GBC	ND*	ND*
	B-M	0.003% (± 0.005%)	0.002% (± 0.003%)

**Table 2:** Mean percent recovery (± standard deviation) and detection frequency of Murine Norovirus (MNV-1) and Murine Hepatitis Virus (MHV-3) from the three meat matrices, using the PEG, GBC, and B-M methods. The “Samples Recovered” column indicates the number of positive replicates out of six total (*n* = 6). ND indicates Not Detected, and ND* indicates recovery < 0.001%

Matrix	Method	MNV-1 Mean % (± SD %)	MHV-3 Mean % (± SD %)	Samples Recovered (MNV-1 | MHV-3)
Chicken	PEG	0.344 (± 0.534)	2.785 (± 3.210)	(2/6 | 6/6)
	GBC	ND	0.011 (± 0.015)	(0/6 | 4/6)
	B-M	29.454 (± 33.059)	0.213 (± 0.130)	(6/6 | 6/6)
Pork	PEG	0.301 (± 0.494)	0.033 (± 0.040)	(2/6 | 5/6)
	GBC	ND	ND	(0/6 | 0/6)
	B-M	6.407 (± 1.705)	0.198 (± 0.231)	(6/6 | 6/6)
Deli Meat	PEG	0.058 (± 0.137)	0.002 (± 0.003)	(6/6 | 6/6)
	GBC	ND*	0.392 (± 0.591)	(6/6 | 6/6)
	B-M	0.012 (± 0.014)	0.001 (± 0.002)	(6/6 | 6/6)

## Results

The overall detection rate of the three methodologies was first assessed across the nine food products (Table 1). A successful detection was defined as a recovery rate ≥ 0.001%. The B-M method recovered in a higher range of samples, detecting MNV-1 in 100% (9/9) and MHV-3 in 88.9% (8/9) of the samples. In contrast, the PEG method achieved detection rates of 55.56% (5/9) for MNV-1 and 77.78% (7/9) for MHV-3. The GBC method performed poorly, failing to detect MNV-1 in any sample (0/9) while recovering MHV-3 in only 33.33% (3/9) of them.

A detailed product-level analysis (Table 1) highlights the percent recovery of GC for each method per product. For MNV-1 recovery, the B-M method consistently yielded higher recovery rates compared to PEG and GBC, yielding the single highest recovery of the study in chicken wing (71.745% ±7.418%) and showed recoveries in other chicken products (breast: 6.500% ±1.075%; thigh: 10.115% ±5.424%). Performance in pork was also markedly uniform (rib chop: 7.085% ±2.864%; belly: 6.830% ±1.485%; ground pork: 5.310% ±0.636%). B-M was the only method to achieve quantifiable recovery (> 0.001%) in all three deli meat products, though at low levels (Italian salami: 0.018% ±0.021%; colonial salami: 0.013% ±0.010%; coppa: 0.003% ±0.005%). In contrast, the PEG method’s performance was sporadic. It recovered MNV-1 from only two chicken products (breast: 0.540% ±0.764%; thigh: 0.490% ±0.693%) and two pork products (rib chop: 0.580% ±0.820%; ground pork: 0.320% ±0.453%). In the deli meat matrix, PEG achieved one quantifiable recovery (coppa: 0.173% ±0.195%) but only trace detection (defined here as a recovery of < 0.001%, denoted as ND*) in the other two products. The GBC method performed most poorly, failing to recover any quantifiable MNV-1 from any chicken or pork product, achieving only trace amplification (< 0.001% recovery, ND*) in all three deli meat products.

Product-level patterns of MHV-3 recovery (Table 1) differed from those of MNV-1, with no single method demonstrating clear better results across all matrices. In the chicken matrix, performance was highly varied. The PEG method yielded the highest individual recoveries (wing: 1.525% ±0.092%; thigh: 6.830% ±0.339%) but failed to recover virus from breast (ND*). In contrast, the B-M method was more consistent, successfully recovering virus from all three products, though at lower rates (wing: 0.175% ±0.049%; breast: 0.370% ±0.014%; thigh: 0.090% ±0.028%). The GBC method was least effective, with > 0.001% recovery in only one of the three chicken products. For the pork matrix, both B-M and PEG recovered MHV-3 from all three products, with B-M presenting slightly higher and more uniform means. The GBC method, once again, achieved only one recovery > 0.001% in this matrix. The deli meat matrix proved challenging and showed a different pattern of success. Here, the GBC method showed its strongest performance, with quantifiable recovery in two products (Italian salami: 0.203% ±0.189%; colonial salami: 0.970% ±0.694%). The B-M and PEG methods also achieved quantifiable recovery in two of the three deli meat products, confirming the difficult nature of this matrix for all protocols.

To assess method recovery across compositional diversity, recovery data were aggregated by matrix (Table 2; Fig. 1). For MNV-1, the aggregated data confirmed the B-M method’s better recovery in the chicken and pork matrices, producing the highest mean recoveries (chicken: 29.454% ±33.059%; pork: 6.407% ±1.705%) (Table 2). The high standard deviation observed in the chicken matrix is directly attributable to the high recovery from wing tissue (71.745%, Table 1), which substantially elevated the aggregated mean. In contrast, the PEG method’s performance was poor and inconsistent, with replicates visually scattered (Fig. 1a), and many at the detection baseline (10^0^ GC), resulting in low mean recoveries across all matrices (chicken: 0.344%; pork: 0.301%; deli meat: 0.058%). The GBC method failed entirely in chicken and pork, with all replicates clustering at the baseline (Fig. 1a) and no quantifiable recovery (Table 2), while presenting a mean recovery of < 0.001% for deli meat.

For MHV-3 (Fig. 1b; Table 2), no single method demonstrated consistent higher recovery yields across all matrices. In chicken, PEG achieved the highest mean recovery (2.785% ±3.210%), while B-M showed lower but more consistent recovery (0.213% ±0.130%). In pork, B-M was most effective (0.198% ±0.231%), outperforming GBC (no detection) and showing comparable performance to PEG (0.033% ±0.040%). The deli meat matrix proved challenging for all methods, with GBC unexpectedly showing the highest mean recovery (0.392% ±0.567%), although with high difference between repetitions.

To assess the statistical differences across methodologies, the data was aggregated into three separate matrices: chicken, pork, and deli meat. All three matrices presented significant results across methods for MNV-1 (chicken: *p* < 0.001; pork: *p* < 0.001; deli meat: *p* = 0.045) and MHV-3 (chicken: *p* = 0.011; pork: *p* < 0.001; deli meat: *p* = 0.015). For MNV-1, Dunn’s test confirmed B-M method higher recovery in both the chicken and pork matrices. For both the chicken and pork matrices, the B-M method yielded significantly higher viral recovery compared to the PEG (*p* = 0.013) and GBC (*p* = 0.001) methods, whereas no significant differences were observed between PEG and GBC (*p* > 0.999). For the deli meat matrix, although the overall Kruskal-Wallis test was significant (*p* = 0.045), Dunn’s post hoc test found no significant pairwise differences (B-M vs. PEG, *p* = 0.091; B-M vs. GBC, *p* = 0.119; PEG vs. GBC, *p* > 0.999).

For MHV-3, results were more varied by matrix. In the chicken matrix, PEG provided significantly higher yields than GBC (*p* = 0.028), but no significant differences were observed between PEG and B-M (*p* > 0.999) or B-M and GBC (*p* = 0.069). In the pork matrix, B-M yielded significantly higher recovery than GBC (*p* = 0.002), which had no detectable recovery. No other comparisons were significant (B-M vs. PEG, *p* = 0.279; PEG vs. GBC, *p* = 0.279). Finally, in the deli meat matrix, while all methods showed low recovery, GBC was significantly more effective than B-M (*p* = 0.017). No significant differences were found between GBC and PEG (*p* = 0.583) or between PEG and B-M (*p* = 0.432).

### Meat products survey

The B-M method, having demonstrated higher consistency and yielding more recovered samples compared to the other two tested methodologies, was selected for a survey of 25 commercial meat products. This survey aimed to detect the presence of NoV, HAV, HEV, RVA, RVC, PAdV, and PCV-2 in these products. Overall, five samples (20%) tested positive for at least one virus, with only HEV and PCV-2 being detected (Table 3). HEV was the most common, found in 16% of samples (4/25). These included three sausage samples (Cq values: 36.79, 37.83, 36.79) and one pork belly (Cq value: 37.18). PCV-2 was found in one sausage sample (4%; Cq value: 37.12). Notably, NoV, HAV, RVA, RVC, and PAdV were not detected in any of the products. All positive samples came from pork, with processed sausages accounting for four of the five detections.

**Table 3:** Prevalence of viruses detected in 25 commercial meat products. The table shows the number of positive samples for each target virus and the products in which they were identified. ND indicates Not Detected

Virus	Detection	Product
Hepatitis E	4/25	Sausage (3); Pork belly (1)
Porcine Circovirus	1/25	Sausage (1)
Rotavirus A	ND	-
Rotavirus C	ND	-
Hepatitis A	ND	-
Norovirus	ND	-
Porcine Adenovirus	ND	-

## Discussion

### Matrix-specific recovery patterns

The aggregation of recovery data by matrix type (chicken, pork, and deli meat) was designed to provide a comprehensive assessment of each method’s efficiency across compositionally diverse products within the same general category. This approach simulates real-world monitoring scenarios where protocols must perform reliably across products with varying organoleptic properties such as different fat content, moisture levels, and protein compositions within a single surveillance program. For the non-enveloped MNV-1, the B-M method consistently outperformed PEG and GBC in both raw chicken and pork matrices. The higher recovery observed in specific poultry cuts, such as the chicken wing, likely reflects the lower fat content and higher moisture availability in these tissues, which facilitates viral elution. In contrast, the poor and inconsistent performance of the PEG and GBC methods, where most replicates clustered at the detection baseline, highlights their inadequacy for non-enveloped virus recovery in these raw matrices.

The recovery efficiencies observed in this study for MNV-1 (B-M: ~29% in chicken, ~ 6% in pork) are consistent with or exceed benchmarks reported in similar food matrix evaluations. For instance, TRIzol-based methods have yielded recovery efficiencies of 50.1% for NoV GII in ham, though efficiency drops significantly in other matrices [27]. Similarly, an optimized PEG-based methodology followed by automated extraction demonstrated broad recovery ranges, from 2.83% to 35.97% for NoV GII, across various ready-to-eat meats [28]. Furthermore, HEV recovery rates of 34.1% have been reported in pork meat using a protocol that optimized homogenization with TRIzol and spin-column extraction [29]. While the B-M method consistently recovered MNV-1 across specific matrices, these comparative data confirm that no single extraction protocol is universally optimal, and that recovery depends heavily on the specific interplay between virus genotype and matrix physicochemical properties [27–29].

For the enveloped MHV-3, no single method demonstrated consistently higher recovery yields, indicating a matrix-dependent recovery profile. While the PEG method was most effective in chicken tissues, the B-M method proved more reliable for pork. Interestingly, the deli meat matrix was universally challenging for all protocols, with the GBC method performing better than B-M. This matrix-dependent performance variation likely reflects the complex physicochemical composition of processed meats, including high fat content, low moisture, and the presence of additives and preservatives that differentially affect viral elution and recovery [4].

### Concentration method’s strengths and limitations

The higher recovery of the B-M method compared to PEG and GBC can be attributed to several key protocol characteristics. β-mercaptoethanol functions as a reducing agent that disrupts disulfide bonds in proteins, which can facilitate the release of viral particles from the food matrix while simultaneously reducing the co-extraction of PCR inhibitors such as myoglobin and hemoglobin [4, 30]. The protocol’s short execution time and straightforward procedure enhance reproducibility and make it particularly suitable for high-throughput surveillance programs requiring the analysis of large sample volumes by multiple laboratories without requiring extensive technical expertise.

However, an important methodological consideration is that in the B-M protocol, the process control was added at a later step (to a 0.25 g aliquot of homogenate), while in the PEG and GBC methods, controls were added to the initial sample. This difference means that B-M’s higher recovery rates may be partially attributable to reduced exposure of the control virus to initial homogenization processes, which could result in artificial inflation of recovery percentages. This limitation should be considered when interpreting the absolute recovery values, though the method’s consistent performance across diverse matrices remains a notable advantage. While this limitation restricts direct comparisons of the absolute recovery efficiencies, the B-M method’s consistent capacity to yield quantifiable viral RNA across compositionally diverse matrices, and its successful detection of natural contamination in the commercial survey, remains a reliable and operationally significant finding.

Despite its advantages, the B-M method employs a lysis buffer containing β-mercaptoethanol, which prevents the recovery of viable viral particles. This combination functions as a strong reducing and denaturing environment that actively disrupts disulfide bonds and denatures viral structural proteins, effectively destroying the viral capsid and envelope. Because it destroys the intact viral particle to release the nucleic acids, this method prevents the recovery of viable viruses. This limitation restricts its application strictly to molecular detection methods (where only naked RNA/DNA is required) and precludes infectivity assays in cell culture [31]. Additionally, β-mercaptoethanol is toxic, requiring appropriate laboratory safety measures and careful handling, which may increase operational risks in routine surveillance settings [32].

Meanwhile, the PEG method has been utilized for decades in viral concentration protocols across various matrices [16] and operates by binding water molecules to precipitate proteins and associated viral particles [30]. While the results showed inconsistent recovery for MNV-1 across meat matrices, PEG demonstrated good performance for MHV-3 in certain products, particularly in chicken wing and thigh tissues (Fig. 1). A significant advantage of the PEG method is its capacity to recover viable viral particles, making it suitable for subsequent cell culture infectivity assays [33]. However, in meat products, PEG also precipitates PCR inhibitors including myoglobin and hemoglobin, which can interfere with downstream molecular detection [4]. The method’s long execution time, meticulous handling requirements, and high inter-replicate variability (as evidenced by scattered data points at the detection baseline) limit its reproducibility across different laboratories and make it less suitable for high-throughput applications.

Finally, the GBC method performed poorly across most matrices, particularly for non-enveloped virus recovery (Fig. 1). This poor performance likely stems from the substitution of TRI Reagent^®^ with 0.25 M glycine buffer (pH 9.5) in the elution step, which is a modification implemented to reduce costs. The original protocol described by Moor and collaborators 2018 achieved higher recovery rates using TRI Reagent^®^, suggesting that this substitution significantly compromised method performance. Interestingly, the GBC method showed its strongest performance in the deli meat matrix for MHV-3 recovery, significantly outperforming B-M (Fig. 1). This unexpected result may relate to the use of chloroform in the protocol, which could more effectively remove the high lipid content characteristic of processed meats. However, the use of chloroform inactivates enveloped viruses and renders the eluate cytotoxic, precluding any subsequent cell culture-based infectivity assessments [34, 35].

### Matrix composition effects on viral recovery

Chicken products generally yielded higher viral recoveries compared to pork and deli meat, likely due to lower fat concentrations in poultry tissues. Fat content poses a significant challenge for viral recovery from food matrices, as lipids can sequester viral particles and co-precipitate during concentration steps, thereby reducing elution efficiency [36]. Deli meats presented the most challenging matrix for all methods, with the lowest recovery rates across both viral models. The combination of high fat content, low moisture availability, and the presence of curing salts, preservatives, and other additives creates a particularly hostile environment for viral elution and detection. The reduced moisture content in these processed products increases viral particle absorption into the tissue matrix, substantially hindering elution efficiency [36].

### Impacts of meat product survey

Using the B-M method, viral nucleic acids were detected in 20% (5/25) of commercial meat products, with HEV (16%) and PCV-2 (4%) being the only viruses identified. All positive samples originated from pork products, with processed sausages accounting for four of five detections. The absence of NoV, HAV, RVA, RVC, and PAdV in any samples is noteworthy, though the relatively small sample size limits definitive conclusions about their prevalence. The detection of HEV genetic material in pork products has significant public health implications under a One Health framework [37, 38]. HEV represents a zoonotic pathogen with the ability to infect both humans and animals, and pork products are recognized as important transmission vehicles [4]. Similarly, PCV-2, while primarily a swine pathogen causing post-weaning multisystemic wasting syndrome, has implications for animal health surveillance and biosecurity. The high Cq values (36.79–37.83 for HEV; 37.12 for PCV-2) indicate relatively low viral genomic loads in the positive samples. It is crucial to note that molecular detection does not discriminate between infectious viral particles and non-viable nucleic acids; therefore, these positive qPCR results do not confirm viral infectivity. However, the presence of these genomes remains highly relevant. Even if the detected viral particles were inactivated during processing, their presence in retail products serves as a critical bio-indicator of HEV and PCV-2 circulation within swine herds, representing a significant concern for agricultural biosecurity and animal health. Furthermore, given the characteristically low infectious doses of many foodborne viruses [39], the presence of viral genomes underscores a potential transmission risk and highlights the need for continued surveillance.

In the present study, the rate of 16% in commercial retail products closely mirrors the HEV prevalence rates consistently observed at the primary production level across both regional and global surveillance studies. Within the South American context, a previous study detected HEV-3 RNA in 15.2% of bile samples from healthy slaughtered pigs in Brazil [40], while another survey reported HEV RNA in 16.6% of domestic pig stool samples and 10.8% of untreated wastewater in Uruguay [41]. These regional findings are corroborated by large-scale European studies; Italian surveillance detected HEV RNA in 14.5% of pooled fecal samples from local farms [42], and similar research reported a 10.8% detection rate in Bulgarian farrow-to-finish operations [43]. Both European studies highlighted that fattener pigs exhibit the highest shedding rates (up to 20.6%) immediately prior to slaughter [3, 4]. Interestingly, while environmental surveillance at the slaughterhouse level often yields low or null detection rates, as demonstrated by a recent study that detected no HEV RNA across 45 critical environmental sampling points in Italian abattoirs [44], the virus clearly persists in the food production chain. Therefore, the 16% molecular positivity rate observed in retail sausage and pork belly samples in the present study suggests that the viral load harbored internally by asymptomatically infected pigs at slaughter successfully bypasses standard industrial processing hurdles, translating into persistent molecular contamination at the final consumer retail level.

Previous surveys highlight a discrepancy in HEV positivity depending on sausage formulation [17, 37, 45, 46]. Commercial sausages lacking liver tissue generally exhibit negligible or extremely low HEV RNA prevalence, ranging from 0% in Canadian and Swiss raw pork sausages [17, 37, 45] to 1.75%–2% in Irish fresh and fermented sausages [46]. Higher detection rates (11.8% to 18.9%) are typically restricted to sausages formulated with pooled high-risk tissues, such as liver [17, 45]. The sausages evaluated in our study were formulated exclusively with muscle meat, making our 16% detection rate notably higher than the established rate for non-liver sausages. This elevated prevalence can likely be attributed to several converging factors. The observed contamination levels may mirror a high regional HEV herd seroprevalence within the source animal populations [40]. Furthermore, the blending of meat during manufacturing, coupled with cross-contamination from plant surfaces and retail tools, can broadly disseminate the virus from infected animals to standard muscle-based products [17, 37]. Ultimately, the discrepancy between our findings and the lower rates reported in meat-only sausages elsewhere may be due to the viral recovery methodology.

Finally, it is important to acknowledge the limitations inherent to this specific study setting. The epidemiological survey was geographically restricted to commercial products purchased within the state of Santa Catarina, Brazil. While this region represents a highly relevant pole for meat production and export, the localized sampling frame and the relatively small sample size (*n* = 25) restrict the ability to extrapolate these specific viral prevalence rates to a national or global scale. Consequently, these findings should be interpreted as a critical baseline indicator of viral circulation within this specific supply chain rather than a comprehensive epidemiological assessment. Future public health surveillance models should aim to expand the geographic sampling radius and increase the statistical power of the survey to build a more reliable predictive understanding of foodborne viral risks.

### Methodological challenges and limitations

Several inherent challenges affect viral recovery from complex food matrices. The low infectious doses of foodborne viruses, often fewer than 100 viral particles, necessitate highly efficient concentration methods [4]. The high concentration of organic and inorganic PCR inhibitors in meat products, including myoglobin, hemoglobin, bile salts (in liver products), and lipids, further complicates detection [4, 47]. These factors collectively create scenarios where surveillance programs may yield false-negative results despite the presence of infectious viral particles.

An additional methodological consideration when interpreting these results is the wide variation in the initial sample mass processed by each protocol: 25 g for PEG, 7 g for GBC, and 5 g for B-M, of which a 0.25 g aliquot was carried forward for final nucleic acid extraction. The starting amount of food has significant implications for viral detection in complex matrices. Larger sample volumes, such as the 25 g used in the PEG method, theoretically enhance the probability of detecting low-level, non-homogeneously distributed viral contamination. However, processing larger meat samples simultaneously increases the co-extraction of PCR inhibitors, such as lipids and myoglobin, which can heavily compromise downstream amplification [48]. Conversely, the B-M protocol’s smaller 0.25 g final extraction aliquot reduces the overall inhibitor load and reagent volumes at the point of extraction, favoring downstream molecular detection, even though its initial 5 g starting mass is comparable to that of GBC. To address the inherent risk of poor sample representativeness associated with extracting such small volumes, the modified B-M protocol introduced a pre-homogenization step using 5 g of meat. It is important to note that while this pre-homogenization step improves the overall homogeneity and representativeness of the subsample, it does not necessarily increase viral recovery efficiency. Instead, it represents a necessary methodological compromise to balance the need for representative sampling with the requirement to minimize inhibitor carryover.

Another limitation of the current study is the absence of an empirically derived LOD, such as an LOD_50_, which requires spiking serial dilutions of the virus directly into the matrix. However, a theoretical minimum recoverable viral load per gram of tissue can be estimated using the operational parameters of the B-M method and the analytical limits of RT-qPCR assay. Based on a minimum analytical detection limit of 10 GC/µL in the PCR template, a tenfold dilution of the nucleic acids prior to amplification, and the specific volume transfers during extraction of the 0.25 g aliquot (200 µL input from a 1 mL homogenate, yielding a 50 µL final eluate), approximately 10^5^ GC must be recovered per gram of tissue to ensure reliable detection. It is important to note that this theoretical calculation assumes 100% extraction efficiency.

An important limitation of process control studies is that artificially inoculated viruses may not accurately reflect the recovery of viruses in naturally contaminated samples. Different viruses possess distinct physical and chemical properties, including variable isoelectric points, which influence viral particle adhesion to and elution from food matrices [48]. For example, human norovirus GII binds strongly within oyster digestive tissue, making elution and detection more challenging than for surrogate viruses [49]. Despite this limitation, the detection of HEV and PCV-2 in naturally contaminated commercial samples demonstrates that the B-M method can overcome the barriers associated with natural contamination, not merely those of experimental inoculation studies.

The use of a Stomacher-type homogenizer, while ensuring protocol reproducibility across laboratories with standard microbiological equipment [50], showed reduced efficiency for low-moisture matrices such as deli meats. Regarding the downstream processing, a single, standardized magnetic bead-based nucleic acid extraction protocol was utilized for all samples. Standardizing this step effectively eliminated extraction efficiency as a confounding variable, ensuring that the significant differences observed in viral recovery can be confidently attributed to the varying efficiencies of the upstream concentration protocols (PEG, GBC, and B-M). However, it is worth noting that while magnetic bead extraction is rapid and cost-effective for high-throughput screening, it may yield lower absolute nucleic acid recoveries compared to silica column-based extraction, particularly in complex food matrices [51].

## Conclusions and recommendations

The B-M method demonstrated the highest consistency and detection rates for non-enveloped viruses across diverse meat matrices, making it a suitable option for large-scale molecular surveillance programs where viable virus recovery is not required. Its short execution time, ease of use, and reproducibility may offset the disadvantages of toxicity and inability to recover infectious particles. For applications requiring viability assessment, the PEG method remains a viable alternative despite its inconsistent performance and longer processing time. Furthermore, the detection of HEV and PCV-2 in commercial pork products underscores the importance of continued surveillance at the food production level. Future research should focus on expanding sample sizes, evaluating seasonal and geographic variations in viral prevalence, and developing rapid, field-deployable detection methods that maintain the sensitivity achieved by laboratory-based protocols. Additionally, optimization of protocols for challenging matrices such as processed meats, potentially through enhanced lipid removal or alternative elution buffers, would improve detection capabilities and reduce false-negative rates in routine monitoring programs.

## Data Availability

The data that support the findings of this study are available from the corresponding author upon reasonable request.

## References

[1] Todd ECD (2014) Foodborne diseases: overview of biological hazards and foodborne diseases. Encyclopedia of food safety. Elsevier, pp 221–242

[2] Miranda RC, Schaffner DW (2019) Virus risk in the food supply chain. Curr Opin Food Sci 30:43–48. 10.1016/j.cofs.2018.12.002

[3] Mortari A, Kolling D, Sobral D, Kist A, De Dea Lindner J, Fongaro G, Miotto M (2023) Norovirus and rotavirus in surface, malacoculture, and human consumption water in Santa Catarina State, Brazil. J Water Health 21:35–46. 10.2166/wh.2022.188 36705496 10.2166/wh.2022.188

[4] Schrader C, Schielke A, Ellerbroek L, Johne R (2012) PCR inhibitors - occurrence, properties and removal. J Appl Microbiol 113:1014–1026. 10.1111/j.1365-2672.2012.05384.x 22747964 10.1111/j.1365-2672.2012.05384.x

[5] Stals A, Baert L, Van Coillie E, Uyttendaele M (2012) Extraction of food-borne viruses from food samples: A review. Int J Food Microbiol 153:1–9. 10.1016/j.ijfoodmicro.2011.10.014 22137685 10.1016/j.ijfoodmicro.2011.10.014

[6] Kotwal G, Cannon JL (2014) Environmental persistence and transfer of enteric viruses. Curr Opin Virol 4:37–43. 10.1016/j.coviro.2013.12.003 24413147 10.1016/j.coviro.2013.12.003

[7] Bosch A, Gkogka E, Le Guyader FS, Loisy-Hamon F, Lee A, Van Lieshout L, Marthi B, Myrmel M, Sansom A, Schultz AC, Winkler A, Zuber S, Phister T (2018) Foodborne viruses: Detection, risk assessment, and control options in food processing. Int J Food Microbiol 285:110–128. 10.1016/j.ijfoodmicro.2018.06.001 30075465 10.1016/j.ijfoodmicro.2018.06.001 PMC7132524

[8] Nemes K, Persson S, Simonsson M (2023) Hepatitis A Virus and Hepatitis E Virus as Food- and Waterborne Pathogens—Transmission Routes and Methods for Detection in Food. Viruses 15:1725. 10.3390/v15081725 37632066 10.3390/v15081725 PMC10457876

[9] International Organization for Standardization (2017) Microbiology of the food chain—horizontal method for determination of hepatitis A virus and norovirus using real-time RT-PCR—part 1: method for quantification (ISO 15216-1:2017)

[10] Hrdy J, Vasickova P (2022) Virus detection methods for different kinds of food and water samples – The importance of molecular techniques. Food Control 134:108764. 10.1016/j.foodcont.2021.108764

[11] Jia M, Taylor TM, Senger SM, Ovissipour R, Bertke AS (2022) SARS-CoV-2 Remains Infectious on Refrigerated Deli Food, Meats, and Fresh Produce for up to 21 Days. Foods 11:286. 10.3390/foods11030286 35159438 10.3390/foods11030286 PMC8834215

[12] Amoros M, Simõs CMO, Girre L, Sauvager F, Cormier M (1992) Synergistic Effect of Flavones and Flavonols Against Herpes Simplex Virus Type 1 in Cell Culture. Comparison with the Antiviral Activity of Propolis. J Nat Prod 55:1732–1740. 10.1021/np50090a003 1338212 10.1021/np50090a003

[13] Campos -MM, Campos-C R (2017) Applications of quartering method in soils and foods. Int J Eng Res Appl 7:35–39. 10.9790/9622-0701023539

[14] Schlindwein A, Simões C, Barardi C (2009) Comparative study of two extraction methods for enteric virus recovery from sewage sludge by molecular methods. Mem Inst Oswaldo Cruz 104:576–579. 10.1590/S0074-02762009000400007 19722079 10.1590/s0074-02762009000400007

[15] Viancelli A, Garcia LAT, Kunz A, Steinmetz R, Esteves PA, Barardi CRM (2012) Detection of circoviruses and porcine adenoviruses in water samples collected from swine manure treatment systems. Res Vet Sci 93:538–543. 10.1016/j.rvsc.2011.07.022 21872287 10.1016/j.rvsc.2011.07.022

[16] Lewis GD, Metcalf TG (1988) Polyethylene glycol precipitation for recovery of pathogenic viruses, including hepatitis A virus and human rotavirus, from oyster, water, and sediment samples. Appl Environ Microbiol 54:1983–1988. 10.1128/aem.54.8.1983-1988.1988 2845860 10.1128/aem.54.8.1983-1988.1988 PMC202790

[17] Moor D, Liniger M, Baumgartner A, Felleisen R (2018) Screening of Ready-to-Eat Meat Products for Hepatitis E Virus in Switzerland. Food Environ Virol 10:263–271. 10.1007/s12560-018-9340-x 29492902 10.1007/s12560-018-9340-x PMC6096950

[18] Degenhardt R, Sobral Marques Souza D, Acordi Menezes LA, De Melo Pereira GV, Rodríguez-Lázaro D, Fongaro G, De Dea Lindner J (2021) Detection of Enteric Viruses and Core Microbiome Analysis in Artisanal Colonial Salami-Type Dry-Fermented Sausages from Santa Catarina, Brazil. Foods 10:1957. 10.3390/foods10081957 34441733 10.3390/foods10081957 PMC8392621

[19] Baert L, Uyttendaele M, Van Coillie E, Debevere J (2008) The reduction of murine norovirus 1, B. fragilis HSP40 infecting phage B40-8 and E. coli after a mild thermal pasteurization process of raspberry puree. Food Microbiol 25:871–874. 10.1016/j.fm.2008.06.002 18721675 10.1016/j.fm.2008.06.002

[20] Besselsen DG, Wagner AM, Loganbill JK (2002) Detection of rodent coronaviruses by use of fluorogenic reverse transcriptase-polymerase chain reaction analysis. Comp Med 52:111–116 12022389

[21] Kageyama T, Kojima S, Shinohara M, Uchida K, Fukushi S, Hoshino FB, Takeda N, Katayama K (2003) Broadly Reactive and Highly Sensitive Assay for Norwalk-Like Viruses Based on Real-Time Quantitative Reverse Transcription-PCR. J Clin Microbiol 41:1548–1557. 10.1128/JCM.41.4.1548-1557.2003 12682144 10.1128/JCM.41.4.1548-1557.2003 PMC153860

[22] Jothikumar N, Cromeans TL, Hill VR, Lu X, Sobsey MD, Erdman DD (2005) Quantitative Real-Time PCR Assays for Detection of Human Adenoviruses and Identification of Serotypes 40 and 41. Appl Environ Microbiol 71:3131–3136. 10.1128/AEM.71.6.3131-3136.2005 15933012 10.1128/AEM.71.6.3131-3136.2005 PMC1151802

[23] Jothikumar N, Cromeans TL, Robertson BH, Meng XJ, Hill VR (2006) A broadly reactive one-step real-time RT-PCR assay for rapid and sensitive detection of hepatitis E virus. J Virol Methods 131:65–71. 10.1016/j.jviromet.2005.07.004 16125257 10.1016/j.jviromet.2005.07.004

[24] Zeng S-Q, Halkosalo A, Salminen M, Szakal ED, Puustinen L, Vesikari T (2008) One-step quantitative RT-PCR for the detection of rotavirus in acute gastroenteritis. J Virol Methods 153:238–240. 10.1016/j.jviromet.2008.08.004 18765254 10.1016/j.jviromet.2008.08.004

[25] Hundesa A, De Maluquer C, Albinana-Gimenez N, Rodriguez-Manzano J, Bofill-Mas S, Suñen E, Rosina Girones R (2009) Development of a qPCR assay for the quantification of porcine adenoviruses as an MST tool for swine fecal contamination in the environment. J Virol Methods 158:130–135. 10.1016/j.jviromet.2009.02.006 19428581 10.1016/j.jviromet.2009.02.006

[26] Opriessnig T, Yu S, Gallup JM, Evans RB, Fenaux M, Pallares F, Thacker EL, Brockus CW, Ackermann MR, Thomas P, Meng XJ, Halbur PG (2003) Effect of Vaccination with Selective Bacterins on Conventional Pigs Infected with Type 2 Porcine Circovirus. Vet Pathol 40:521–529. 10.1354/vp.40-5-521 12949409 10.1354/vp.40-5-521

[27] Da Silva Luz I, Miagostovich MP (2018) Comparison of viral elution-concentration methods for recovering noroviruses from deli meats. J Virol Methods 260:49–55. 10.1016/j.jviromet.2018.07.001 29981297 10.1016/j.jviromet.2018.07.001

[28] Hennechart-Collette C, Fourniol L, Fraisse A, Perelle S (2026) Characterization of a method to detect hepatitis A virus and norovirus in meat products. Int J Food Microbiol 459:111928. 10.1016/j.ijfoodmicro.2026.111928 42385281 10.1016/j.ijfoodmicro.2026.111928

[29] Di Cola G, Fantilli AC, Rodríguez-Lombardi G, Rucci KA, Castro G, Mirazo S, Nates SV, Pisano MB, Ré VE (2025) Assessment of Hepatitis E Virus RNA Detection in Meat Samples: Optimization of Pre-analytical Conditions. Food Environ Virol 17:1. 10.1007/s12560-024-09617-z 10.1007/s12560-024-09617-z 39580366

[30] Goldring JPD (2019) Concentrating proteins by salt, polyethylene glycol, solvent, SDS precipitation, three-phase partitioning, dialysis, centrifugation, ultrafiltration, lyophilization, affinity chromatography, immunoprecipitation or increased temperature for protein isolation, drug interaction, and proteomic and peptidomic evaluation. In: Kurien BT, Scofield RH (eds) Electrophoretic separation of proteins. Springer New York, pp 41–59 10.1007/978-1-4939-8793-1_4 30426405

[31] Blow JA, Mores CN, Dyer J, Dohm DJ (2008) Viral nucleic acid stabilization by RNA extraction reagent. J Virol Methods 150:41–44. 10.1016/j.jviromet.2008.02.003 18387678 10.1016/j.jviromet.2008.02.003

[32] Sigma-Aldrich (2024) Safety data sheet: β-mercaptoethanol (product no. 516732)

[33] Da Silva VC, Elois M, Savi BP, Miotto M, De Dea Lindner J, Fongaro G, Souza DSM (2023) Bioaccumulation Dynamic by Crassostrea gigas Oysters of Viruses That Are Proposed as Surrogates for Enteric Virus Contamination in Environmental Samples. Food Environ Virol 15:1–7. 10.1007/s12560-022-09538-9 36287375 10.1007/s12560-022-09538-9

[34] Feinstone SM, Mihalik KB, Kamimura T, Alter HJ, London WT, Purcell RH (1983) Inactivation of hepatitis B virus and non-A, non-B hepatitis by chloroform. Infect Immun 41:816–821. 10.1128/iai.41.2.816-821.1983 6409813 10.1128/iai.41.2.816-821.1983 PMC264712

[35] El-shenawy NS, Abdel-Rahman MS (1993) The mechanism of chloroform toxicity in isolated rat hepatocytes. Toxicol Lett 69:77–85. 10.1016/0378-4274(93)90148-Q 8356569 10.1016/0378-4274(93)90148-q

[36] Stals A, Baert L, De Keuckelaere A, Van Coillie E, Uyttendaele M (2011) Evaluation of a norovirus detection methodology for ready-to-eat foods. Int J Food Microbiol 145:420–425. 10.1016/j.ijfoodmicro.2011.01.013 21333370 10.1016/j.ijfoodmicro.2011.01.013

[37] Mykytczuk O, Harlow J, Bidawid S, Corneau N, Nasheri N (2017) Prevalence and Molecular Characterization of the Hepatitis E Virus in Retail Pork Products Marketed in Canada. Food Environ Virol 9:208–218. 10.1007/s12560-017-9281-9 28197972 10.1007/s12560-017-9281-9 PMC5429394

[38] Elois MA, Da Silva Grisard HB, Rodríguez-Lázaro D, Fongaro G (2024) Challenges and global trends in combating enteric hepatitis. J Gen Virol 105. 10.1099/jgv.0.002059 10.1099/jgv.0.002059 39693132

[39] Teunis PFM, Moe CL, Liu P, Miller E, Lindesmith S, Baric L, Le Pendu RS, Calderon J RL (2008) Norwalk virus: How infectious is it? J Med Virol 80:1468–1476. 10.1002/jmv.21237 18551613 10.1002/jmv.21237

[40] Amorim AR, Mendes GS, Pena GPA, Santos N (2018) Hepatitis E virus infection of slaughtered healthy pigs in Brazil. Zoonoses Public Health 65:501–504. 10.1111/zph.12455 29441690 10.1111/zph.12455

[41] Cancela F, Icasuriaga R, Cuevas S, Hergatacorzian V, Olivera M, Panzera Y, Pérez R, López J, Borzacconi L, González E, Montaldo N, Gaitán M, López-Verges S, Bortagaray V, Victoria M, Colina R, Arbiza J, Berois M, Mirazo S (2023) Epidemiology Update of Hepatitis E Virus (HEV) in Uruguay: Subtyping, Environmental Surveillance and Zoonotic Transmission. Viruses 15:2006. 10.3390/v15102006 37896784 10.3390/v15102006 PMC10612089

[42] Ianiro G, Pavoni E, Aprea G, Romantini R, Alborali GL, D’Angelantonio D, Garofolo G, Scattolini S, De Sabato L, Magistrali CF, Burow E, Ostanello F, Smith RP, Di Bartolo I (2023) Cross-sectional study of hepatitis E virus (HEV) circulation in Italian pig farms. Front Vet Sci 10:1136225. 10.3389/fvets.2023.1136225 37143498 10.3389/fvets.2023.1136225 PMC10151646

[43] Krumova-Valcheva GL, Di Bartolo I, Smith RP, Gyurova E, Mateva G, Milanov M, Dimitrova A, Burow E, Daskalov H (2023) Detection of HEV RNA Using One-Step Real-Time RT-PCR in Farrow-to-Finish Pig Farms in Bulgaria. Pathogens 12:673. 10.3390/pathogens12050673 37242343 10.3390/pathogens12050673 PMC10224082

[44] Ianiro G, Pavoni E, De Sabato L, Monini M, Delibato E, Perrone V, Ostanello F, Niine T, Di Bartolo I (2024) Investigation of Salmonella, hepatitis E virus (HEV) and viral indicators of fecal contamination in four Italian pig slaughterhouses, 2021–2022. Res Vet Sci 171:105209. 10.1016/j.rvsc.2024.105209 38460205 10.1016/j.rvsc.2024.105209

[45] Giannini P, Jermini M, Leggeri L, Nüesch-Inderbinen M, Stephan R (2018) Detection of Hepatitis E Virus RNA in Raw Cured Sausages and Raw Cured Sausages Containing Pig Liver at Retail Stores in Switzerland. J Food Prot 81:43–45. 10.4315/0362-028X.JFP-17-270 29257725 10.4315/0362-028X.JFP-17-270

[46] Bennett C, Coughlan S, Hunt K, Butler F, Fanning S, Ryan E, De Gascun C, O’Gorman J (2024) Detection of hepatitis E RNA in pork products at point of retail in Ireland – Are consumers at risk? Int J Food Microbiol 410:110492. 10.1016/j.ijfoodmicro.2023.110492 37988969 10.1016/j.ijfoodmicro.2023.110492

[47] Harrison L, Ramos TDM, Wu X, DiCaprio E (2021) Presence of hepatitis E virus in commercially available pork products. Int J Food Microbiol 339:109033. 10.1016/j.ijfoodmicro.2020.109033 33401188 10.1016/j.ijfoodmicro.2020.109033

[48] Michen B, Graule T (2010) Isoelectric points of viruses. J Appl Microbiol 109:388–397. 10.1111/j.1365-2672.2010.04663.x 20102425 10.1111/j.1365-2672.2010.04663.x

[49] Maalouf H, Schaeffer J, Parnaudeau S, Le Pendu J, Atmar RL, Crawford SE, Le Guyader FS (2011) Strain-Dependent Norovirus Bioaccumulation in Oysters. Appl Environ Microbiol 77:3189–3196. 10.1128/AEM.03010-10 21441327 10.1128/AEM.03010-10 PMC3126434

[50] Armstrong CM, He Y, Chen C-Y, Counihan K, Lee J, Reed S, Capobianco J (2024) Use of a commercial tissue dissociation system to detect Salmonella-contaminated poultry products. Anal Bioanal Chem 416:621–626. 10.1007/s00216-023-04668-w 37055639 10.1007/s00216-023-04668-w

[51] Sayed IM, Hammam ARA, Elfaruk MS, Alsaleem KA, Gaber MA, Ezzat AA, Salama EH, Elkhawaga AA, El-Mokhtar MA (2020) Enhancement of the Molecular and Serological Assessment of Hepatitis E Virus in Milk Samples. Microorganisms 8:1231. 10.3390/microorganisms8081231 32806687 10.3390/microorganisms8081231 PMC7465259

